# Resection of Biliary Cystadenoma in a Pregnant Woman: A Case Report With Five Years of Follow-Up

**DOI:** 10.7759/cureus.56752

**Published:** 2024-03-22

**Authors:** Patryk Patrzałek, Agnieszka Hałoń, Maciej Guziński, Michał Pomorski, Wojciech Tokarczyk, Dariusz Patrzałek

**Affiliations:** 1 Department of Surgery, District Hospital in Rawicz, Rawicz, POL; 2 Department of Pathomorphology and Oncological Cytology, Wroclaw Medical University, Wroclaw, POL; 3 Department of Radiology, Wroclaw Medical University, Wroclaw, POL; 4 Department of Gynecology, Wroclaw Medical University, Wroclaw, POL; 5 Department of Cardiology, Wroclaw University Hospital, Wroclaw, POL; 6 Department of Vascular, General and Transplant Surgery, Wroclaw Medical University, Wroclaw, POL

**Keywords:** biliary tumors, surgery in pregnancy, biliary cystadenoma, liver cyst, liver surgery, liver tumor

## Abstract

Biliary cystadenomas (BCAs), rare cystic tumors occurring in the biliary system, account for fewer than 5% of cystic lesions in the liver. This case details successful resection in a 29-year-old pregnant woman at seven weeks gestation. Urgent left hemihepatectomy and cholecystectomy removed a mucinous hepatobiliary cystadenoma. Postoperatively, a healthy newborn was delivered by cesarean section. Five-year follow-up showed no recurrence. BCAs present diagnostic challenges due to nonspecific symptoms, and surgical intervention, preferably complete resection, is recommended for potential malignancy, after weighing benefits against complications in critical hepatic vessel lesions.

## Introduction

Biliary cystadenomas (BCAs) are uncommon, complex cystic tumors that can develop within the biliary system of the liver or in the extrahepatic bile ducts, including the gallbladder [1,2]. Liver cysts represent the most prevalent liver lesions, occurring in approximately 20% of the general population. Among all liver cysts, fewer than 5% are classified as BCAs [3]. The lesions were first described by Hueter in Göttingen in 1887 [4]. Since then, approximately 200 cases were reported until 2013, and this number surged to over 970 in 2019 [1,5]. BCAs carry an approximate 20% potential for malignancy [5]. Because of the rarity of BCAs and relatively nonspecific abdominal symptoms, numerous clinicians may lack familiarity with the diagnostic characteristics and therapeutic approaches associated with BCAs. The gold standard for managing this specific diagnosis is complete excision followed by a meticulous histopathological examination. The discovery of a substantial intrahepatic cyst in a patient during the early stages of pregnancy, mandating surgical intervention due to critical indications, introduces an augmented risk profile for both maternal and fetal well-being.

In this case report, we present the successful and complete resection of a BCA in a 29-year-old pregnant woman in her seventh week of gestation, resulting in a healthy newborn.

## Case presentation

**Figure 1:**
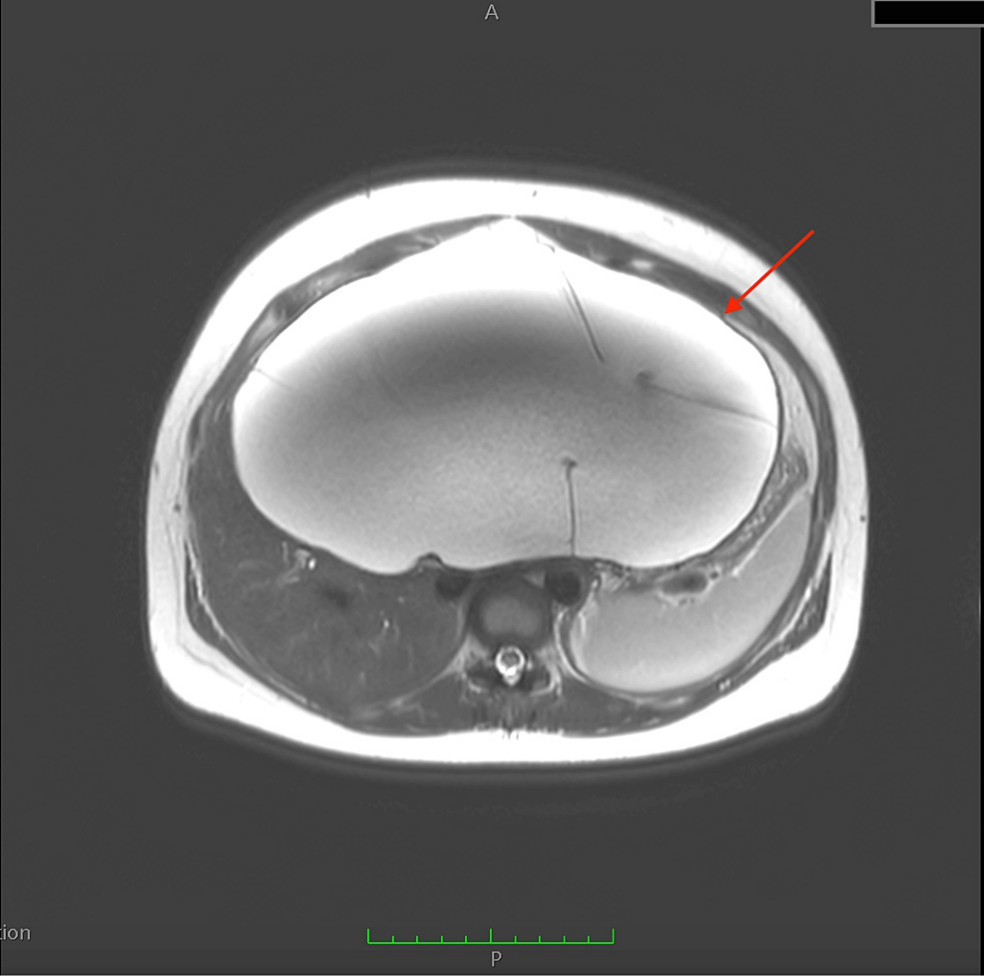
Preoperative MRI. Giant cyst, with a thin wall, in the left part of the liver with multiple septa. The presence of the giant cyst is indicated by the red arrow.

**Figure 2:**
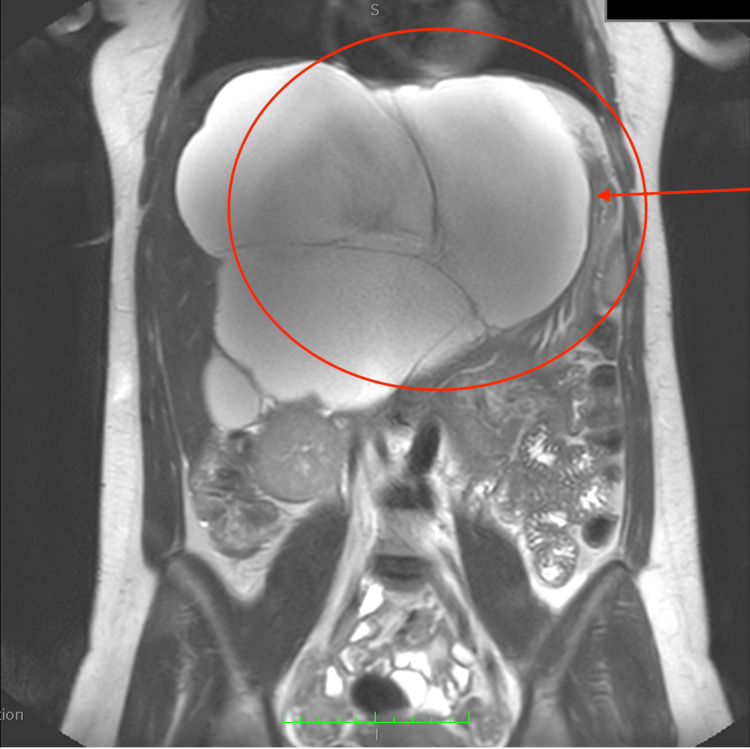
Preoperative MRI. Giant cyst, with a thin wall, in the left part of the liver with multiple septa. The presence of the giant cyst is indicated by the red arrow.

A 29-year-old woman in the seventh week of her first pregnancy presented to the emergency ward with abdominal pain and an enlarged abdomen. She had no significant medical history. Abdominal ultrasound revealed a giant cyst measuring 20.7 × 12 cm and another cyst measuring 7 × 19.8 cm, both with vascularized septa and containing hypoechoic, protein-like fluid. The origin of the tumor was likely the liver. Laboratory tests, including liver function (Child-Pugh A5), were normal. Cancer antigen 19-9 (CA 19-9) level was 2 units, and total beta-human chorionic gonadotropin was 103,503 mlU/mL. MRI confirmed a giant cyst with a thin wall in the left part of the liver, exhibiting multiple septa (Figures 1, 2).

The patient underwent an urgent operation due to life indications. A gynecological consultation confirmed a seven and six/seven-week pregnancy, with a single embryo measuring 14.8 mm present in the uterus. Substitution with progesterone, administered three times at 100 mg per vagina, was prescribed in the preoperative period.

Left hemihepatectomy, involving segments II, III, and IV along with cholecystectomy, was successfully performed with a clear macroscopic surgical margin. The postoperative course was uneventful, and the patient was discharged from the hospital on the 11th postoperative day, including a two-day stay in the intensive care unit.

**Figure 3:**
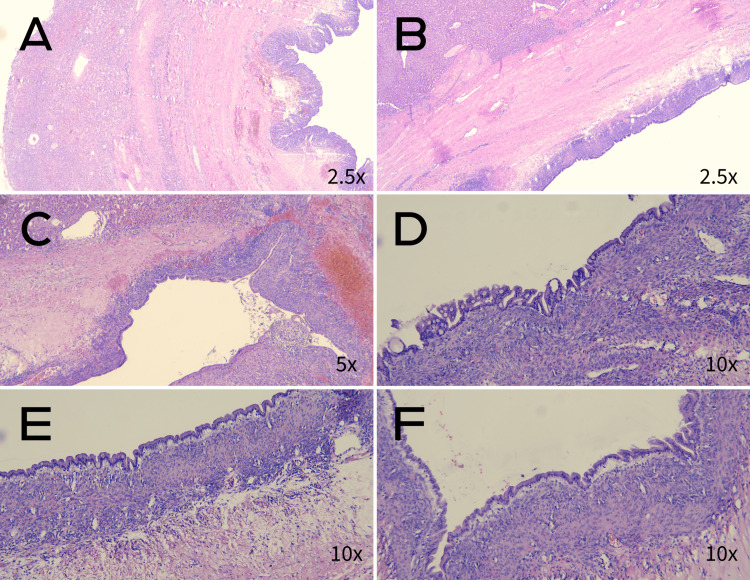
Mucinous hepatobiliary cystadenoma with mesenchymal stroma. A, B, C: liver parenchyma and tumor wall. D, E, F: cylindrical glandular epithelium.

**Figure 4:**
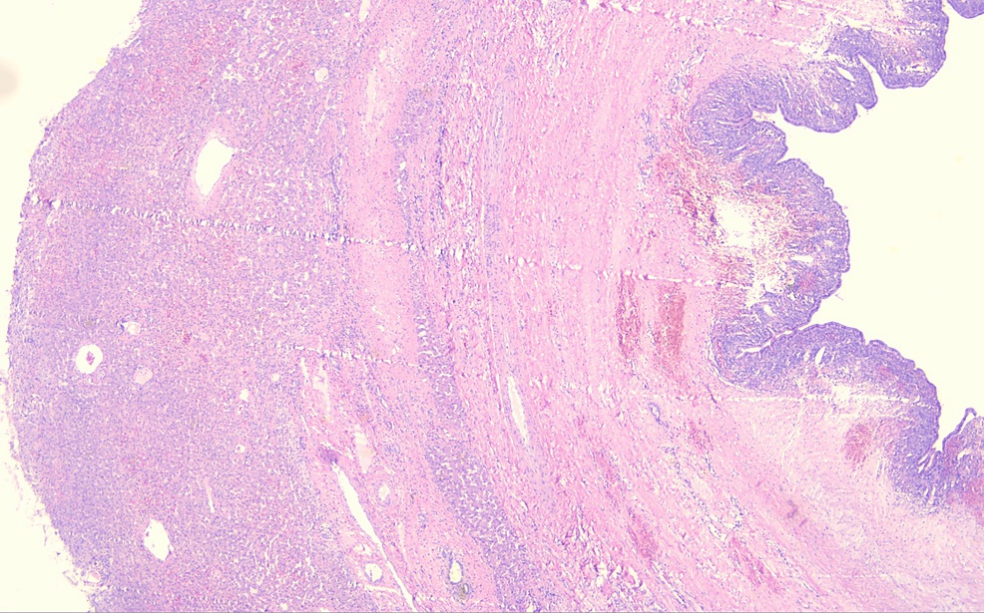
Mucinous hepatobiliary cystadenoma with mesenchymal stroma. Liver parenchyma and tumor wall.

The histopathological examination revealed a mucinous hepatobiliary cystadenoma with mesenchymal stroma (Figures 3, 4). The examination confirmed a complete resection of the benign tumor with a margin of more than 1 cm. Cyst fluid cytology showed bloody liquid with numerous macrophages and no neoplastic cells.

The patient remained under close gynecological observation. The pregnancy was completed on schedule through a cesarean section, resulting in the delivery of a healthy newborn girl. The Apgar score was 10.

**Figure 5:**
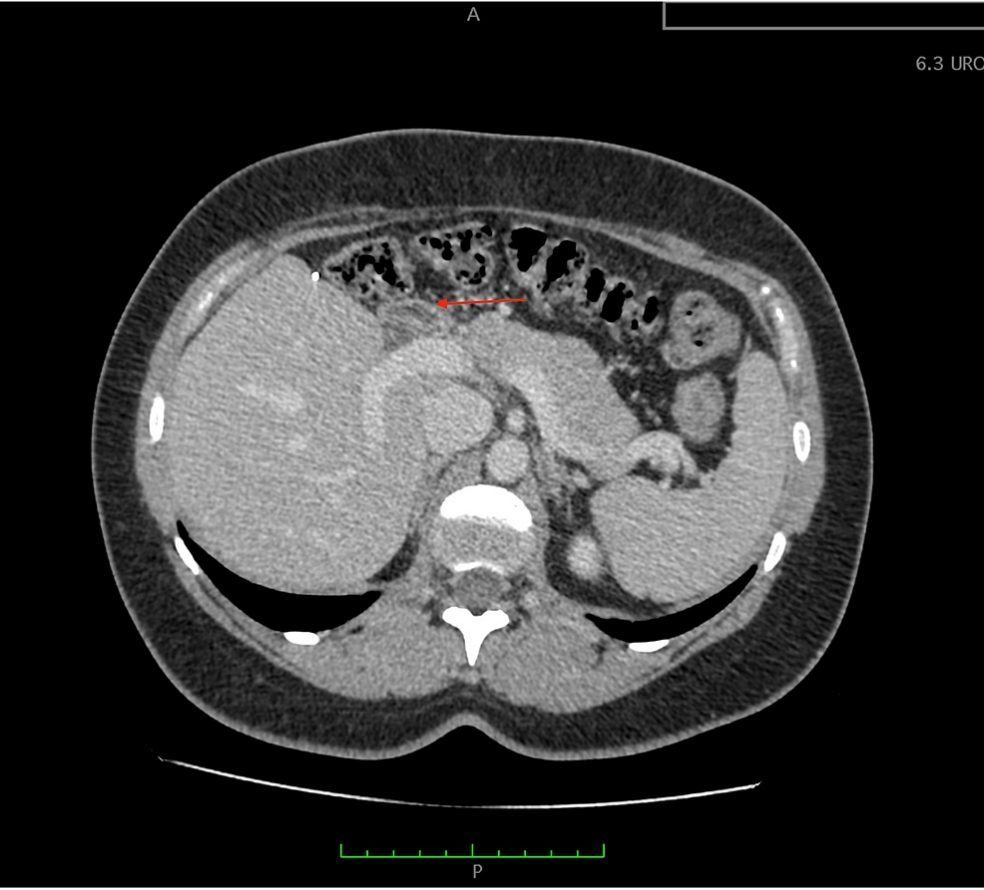
Follow-up CT scan five years after the surgery. Following resection, the postoperative anatomical location (red arrow).

**Figure 6:**
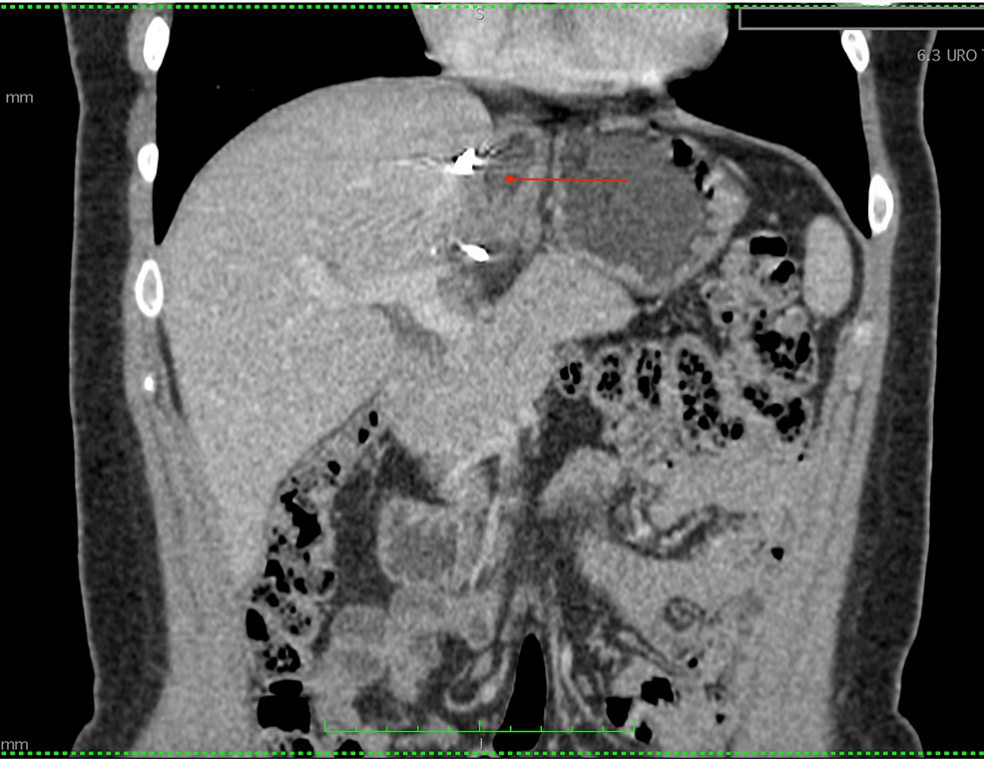
Follow-up CT scan five years after surgery. Following resection, the postoperative anatomical location (red arrow).

Five years post-operation, the patient underwent a follow-up abdominal CT, revealing no signs of recurrence (Figures 5, 6). Her laboratory tests, including oncological markers, were all normal. The pediatrician confirmed that the child’s mental and physical growth exhibited no abnormalities.

## Discussion

BCAs represent a rare category of neoplasms affecting the biliary ducts. Accounting for fewer than 5% of all hepatic cystic lesions [6], BCAs are characterized as benign; nevertheless, they carry an approximate 20% potential for malignancy [5]. Caution is warranted in interpreting these figures due to the absence of established diagnostic criteria.

The clinical manifestations of BCA are nonspecific, encompassing symptoms such as abdominal pain, with reported frequencies ranging from 25.7% [7] to 62% [8]. Additionally, less frequently observed symptoms of BCA include abdominal mass (17.9%), abdominal distension (10.3%), obstructive jaundice (5.1%), and fever (7.7%) [7]. According to the literature, a notable proportion of BCA cases, i.e., 25.4%, are asymptomatic [1].

Women constitute 90.7% of the patients suffering from BCAs. According to the systematic review by Klompenhouwer et al., the age range of the patients is reported to be 38-62 years old [1]. Our patient was notably younger; nevertheless, there have been isolated cases diagnosed in early childhood [9].

Imaging modalities commonly utilized in the diagnosis of BCA include ultrasonography, CT, and MRI. Distinctive images displaying septation (71.3%) and multiloculation (69.4%) should prompt consideration of BCA or biliary cystadenocarcinoma (BCAC) during the diagnostic process. Additional imaging features may encompass wall enhancement (26.6%), mural nodes (17.2%), and calcifications (13.3%) [1]. Despite the valuable insights provided by these imaging tools regarding the cyst’s nature, there is currently no universally effective and standardized method in the medical field for reliably distinguishing between BCA and BCAC lesions through imaging. Given the limited specificity offered by existing imaging modalities, upcoming research efforts should concentrate on enhancing the diagnostic workup. We share the opinion of Klompenhouwer et al. that one potential avenue for improvement involves exploring combinations of CT and MRI with contrast-enhanced ultrasound (CEUS). In our case, due to the pregnancy of our patient, imaging diagnostic possibilities were limited to ultrasonography and MRI.

The diagnostic significance of an increase in cyst fluid carcinoembryonic antigen and CA 19-9 levels for BCA and BCAC remains controversial. Due to the risk of pleural and peritoneal dissemination, routine fine-needle aspiration and core needle biopsy of suspected BCA should be generally avoided [5]. Nonetheless, an elevated CA 19-9 level in cyst fluid can be a helpful marker for distinguishing BCA/BCAC from common simple cysts [7].

Because of the potential for malignant transformation of lesions, the recommended treatment is surgical intervention with a focus on achieving complete resection whenever feasible. Fenestration or marsupialization, if chosen, may have significantly inferior outcomes, especially in cases of BCA recurrence [1]. In very rare instances, liver transplantation was performed as the only treatment option [5,10].

The prognosis for patients with BCA is generally excellent, with a reported extremally low mortality in those who underwent surgery [1]. Nevertheless, considering that BCA is essentially a benign disease, liver resections for such conditions may carry a significant morbidity rate of up to 20% and a mortality rate of up to 1% [11]. Resection of BCA may not be possible in all cases, especially for lesions located near larger vessels, which could pose a risk of serious complications during surgery. Due to the life indications, our patient underwent the surgery. Despite concerns regarding potential pregnancy endangering, the surgical team successfully conducted a complete resection of the lesion with close supervision by gynecologists in the peri- and postoperative periods. Subsequent follow-up confirmed that both the mother and the child tolerated the surgery well, with no signs of recurrence during the five-year observation.

## Conclusions

To our knowledge, this represents the first reported case of BCA resection during early pregnancy in the literature. Performing surgery on a pregnant patient involves inherent risks; nevertheless, meticulous imaging diagnostics and a favorable lesion location enabled an effective operation. With an experienced surgical team and considering the lesion’s specific location, such a procedure holds the potential for success without compromising the well-being of the fetus.

Furthermore, a growing body of literature underscores an increasing frequency in the diagnosis of BCA, emphasizing the need for continued research and medical attention in this area.

## References

[1] Klompenhouwer AJ, Ten Cate DW, Willemssen FE, Bramer WM, Doukas M, de Man RA, Ijzermans JN (2019). The impact of imaging on the surgical management of biliary cystadenomas and cystadenocarcinomas; a systematic review. HPB (Oxford).

[2] Davies W, Chow M, Nagorney D (1995). Extrahepatic biliary cystadenomas and cystadenocarcinoma. Report of seven cases and review of the literature. Ann Surg.

[3] Teoh AY, Ng SS, Lee KF, Lai PB (2006). Biliary cystadenoma and other complicated cystic lesions of the liver: diagnostic and therapeutic challenges. World J Surg.

[4] Hueter C (1887). [A Large Cystoma of the Liver in a Child With Comments About Cystic Diseases of the Liver]. Marburg R Friedrich’s Univ-Buchdr. Published online January 1887.

[5] Soares KC, Arnaoutakis DJ, Kamel I, Anders R, Adams RB, Bauer TW, Pawlik TM (2014). Cystic neoplasms of the liver: biliary cystadenoma and cystadenocarcinoma. J Am Coll Surg.

[6] Zen Y, Fujii T, Itatsu K (2006). Biliary cystic tumors with bile duct communication: a cystic variant of intraductal papillary neoplasm of the bile duct. Mod Pathol.

[7] Chen YW, Li CH, Liu Z, Dong JH, Zhang WZ, Jiang K (2014). Surgical management of biliary cystadenoma and cystadenocarcinoma of the liver. Genet Mol Res.

[8] El-Magd EA, El-Shobari M, Abdelsalam RA, Abbas A, Elmahdy Y, Hamed H (2023). Clinicopathological features and management of biliary cystic tumors of the liver: a single-center experience. Langenbecks Arch Surg.

[9] Tran S, Berman L, Wadhwani NR, Browne M (2013). Hepatobiliary cystadenoma: a rare pediatric tumor. Pediatr Surg Int.

[10] Romagnoli R, Patrono D, Paraluppi G (2011). Liver transplantation for symptomatic centrohepatic biliary cystadenoma. Clin Res Hepatol Gastroenterol.

[11] Dokmak S, Ftériche FS, Borscheid R, Cauchy F, Farges O, Belghiti J (2013). 2012 Liver resections in the 21st century: we are far from zero mortality. HPB (Oxford).

